# Quality of life of patients with hip fracture was better during the COVID-19 period than before, an ancillary study from the HiFIT multicenter study

**DOI:** 10.3389/fpubh.2024.1362240

**Published:** 2024-04-16

**Authors:** Sigismond Lasocki, Xavier Capdevila, Benjamin Bijok, Maria Lahlou-Casulli, Vincent Collange, Nicolas Grillot, Thibault Loupec, Emmanuel Rineau, Maxime Léger, the HiFIT Investigators, CHU Angers, Guillaume BOUHOURS, Sigismond LASOCKI, Adeline LEBAIL, Maxime LEGER, Elsa PAROT-SCHINKEL, Emmanuel RINEAU, Louis RONY, Bruno VIELLE, CHU – Hôpital Lapeyronie Montpellier, Xavier CAPDEVILLA, Thibault LOUPEC, Benjamin MOUNET, Fabien SWISSER, HIA Clermont-Tonnerre, Marc Danguy des Deserts, CHU Nantes, Raphael Cinotti, Nicolas Grillot, Karim Asehnoune, Antoine Roquilly, CHU de Rennes, Hélène Beloeil, Maria Lahlou-Casulli, Lyon Villeurbanne Medipôle, Vincent Collange, Sébastien PARENT, Ramsay Sante, Bertrand DELANNOY, Olivier Desebbe, CHD Vendée, Alexis Duchalais, CHU Poitiers, Bertrand DRUGEON, Jérémy GUENEZAN, CHU Grenoble Alpes, Pierre BOUZAT, Sabine DREVET, Gaetan GAVAZZI, Jules GREZE, CHRU Lille, Benjamin BIJOK, Delphine GARRIGUE, Jean-Stéphane DAVID

**Affiliations:** ^1^Département Anesthésie-Réanimation, CHU Angers, Université d’Angers, Angers, France; ^2^Department of Anesthesiology and Intensive Care Medicine, Lapeyronie University Hospital, Montpellier, France; ^3^Pôle d'anesthésie-réanimation, CHRU de Lille, Lille, France; ^4^Department of Anesthesiology Critical Care Medicine and Perioperative Medicine, Rennes University Hospital and School of Medicine, Rennes, France; ^5^Department of Anesthesiology, Médipole Lyon Villeurbanne, Villeurbanne, France; ^6^Département Anesthesie Reanimation, CHU Nantes, Nantes, France

**Keywords:** COVID-19, hip fracture, older adult, quality of life, EQ5D

## Abstract

**Background:**

The COVID-19 pandemic had a global impact on people life, notably because of lockdown periods. This could particularly affected patients suffering from hip fracture, who could have been more isolated during these periods. We aim at evaluating the impact of the COVID-19 period (including lockdown periods) on quality of life (QOL) in older adult patients 90 days after a surgery for a hip fracture.

**Subject and methods:**

Ancillary study of the prospective randomized controlled HiFIT study. We compared the QOL measured at 90 days after a hip fracture surgery using the EuroQOL-5 dimensions 3 levels (EQ-5D), the Perceived Quality of life (PQOL) and the Instrumental Activities of Daily Living (IADL) in patients included in the Hifit study before and during the COVID-19 pandemic.

**Results:**

The characteristics of the 161 patients included before and of the 213 included during the COVID period (including 122 (57%) during COVID with containment periods and 91 (43%) during COVID without containment periods) were similar (mean age 84 ± 10 years; 282 (75%) women). The majority (81%) of the patients alive at 90 days had returned to their previous place of residence in both periods. During the COVID period, EQ-5D showed better patient pain/discomfort and anxiety/depression levels. The PQOL happiness was not different, with around 81% of the patient being “happy” or “very happy” during the two periods and the IADL was also similar during the two periods. In the multivariate analysis odd ratios of having poorer outcomes were increased before COVID for pain/discomfort (OR 2.38, 95%CI [1.41–4.15], *p* = 0.001), anxiety (OR 1.89 [1.12–3.21], *p* = 0.017) and mobility (1.69 [1.02–2.86], *p* = 0.044).

**Conclusion:**

Patient’s quality of life measured using different scales was not altered during the COVID period compared to before COVID, 90 days after a hip fracture. Surprisingly, the Pain/Discomfort and Anxiety dimensions of the EQ-5D questionnaires were even better during the COVID period.

**Clinical trial registration:**https://clinicaltrials.gov/ (NCT02972294).

## Introduction

During the last 3 years, the COVID-19 pandemic had a global impact on people’s lives, either directly because of the lethality of COVID-19, with at least two million confirmed deaths from COVID-19 in the European Region, according to the world health organization, or through the deprivation of liberty and all the social measures taken to limit the spread of the disease. COVID-19 had a greater impact on the older patients, who suffered from more severe diseases and were particularly at risk of death (1). In addition, all the interventions taken to slow down the spread of the pandemic may also have aggravated the social isolation of these patients (2), when social connection and integration are crucial factors of older adult patient‘s well-being (3).

In France, a general lockdown was decided on March 17th, 2020, for 9 weeks. Then, many measures were taken, including mobility restrictions, curfews, restaurants and cultural areas closing, sanitary or vaccinal pass… It has already been reported that these measures may impact people’s quality of life, particularly older adult patients. Indeed, these patients are often already isolated and could suffer more from isolation due to all the measures taken to limit the pandemic (4, 5). Many authors consider that older age is an obstacle for the “return to normal life,” as older patients are frail and helpless (6).

Older adult patients are also at risk of hip fracture, which are associated with functional impairment, institutionalization and death (7). In this perspective, cognitive functions (7) and social support play an important role for rehabilitation after hip fracture surgery (8). Recovery from hip fracture may thus have been particularly impaired during this pandemic compared to the period before and this may impact the perceived quality of life (QoL) of these patients. However no data are available, to the best of our knowledge.

To contribute to understanding how COVID-19 has affected the lives of older adults in France, and more precisely in patients suffering from hip fractures, we used data from a randomized controlled study on iron and tranexamic acid in patients operated for a hip fracture, the HiFIT study (9, 10), in which we prospectively collected QoL data from different questionnaires. Our aim was to compare hip fracture patients’ quality of life (assessed at 90 days after the hip fracture) after and during the COVID-19 pandemic period.

## Patients and methods

We used the data obtained from the HiFIT study (10). The study protocol has already been published elsewhere (9). Briefly, it is a multicenter (*n* = 13 French public and private hospitals), 2×2 factorial, randomized, double-blinded, controlled trial evaluating the effect of iron isomaltoside vs. placebo and of tranexamic acid vs. placebo on transfusion rate during hospitalization, in patients undergoing emergency surgery for a hip fracture. The study began before the COVID-19 crisis and ended in 2021, during the COVID-19 pandemic, allowing us to compare how the patients rated their QoL before and during COVID-19 periods.

This study was approved by an ethics committee (Comité de Protection des Personnes Ouest II, number 2016/42, approval date November 15, 2016), by the Agence Nationale de Sécurité du Médicament (ANSM, Number 160828A-21, approval date 12/27/2016), by the “Commission Nationale Informatique et Liberté” (CNIL, decision DR-2017-390, approval date 12/14/2017) and by the “Comité Consultatif sur le Traitement de l’Information en matière de Recherche sans le domaine de la Santé” (CCTIRS, Number 16–716, approval date September 29, 2016). All the patients or a next of kin gave a written consent. The trial has been registered on clinicaltrial.gov (NCT02972294) on November 23, 2016 and inclusions started on March 31, 2017. The study was stopped after the first interim analysis, and the last follow-up was done on September 16th, 2021. An amendment was obtained on December 17th, 2020 to collect the COVID status of the patients (defined as COVID positive or not, if a PCR test was positive), at the different study visits.

**Figure 1:**
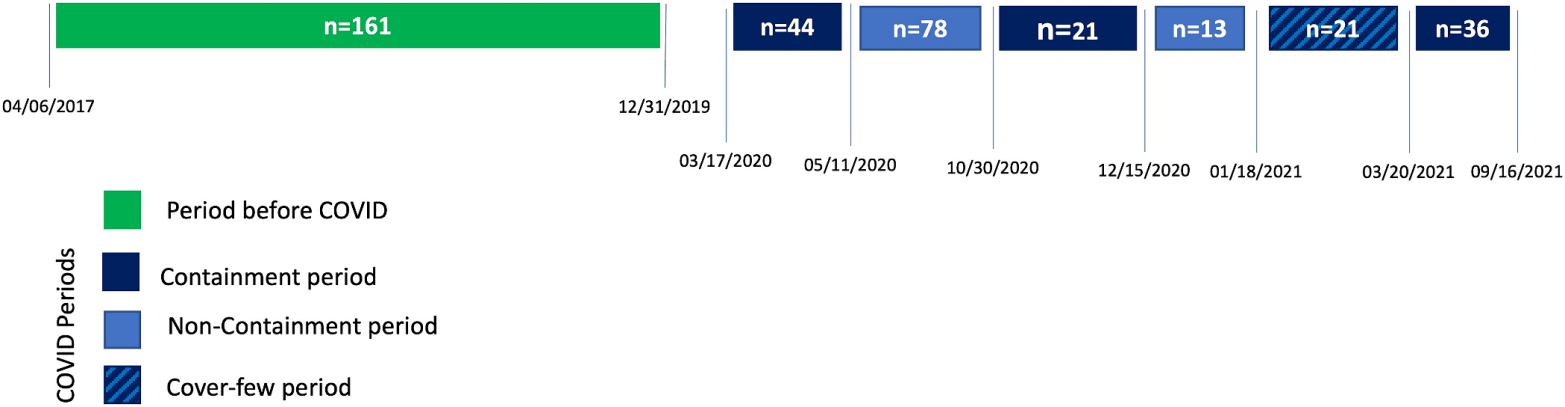
Timeline of the different periods.

For this ancillary study, we analyzed all the patients included in the 2.5-years before COVID (from the April 06, 2017 till the December 31, 2019, *n* = 161 patients) and those included during the COVID period (from the June 03, 2020 till the September 16, 2021, *n* = 213 patients). During the “COVID period,” we identified the periods with liberty restriction (either lockdown or cover-few, defined as “containment periods” see Figure 1) and the periods without restrictions, other than the social distancing and the facial-mask obligations (defined as “non-containment periods”).

### Study population

Patients hospitalized for a hip fracture surgery were eligible for the HiFIT study if they had an osteoporotic hip fracture and preoperative hemoglobin between 9.5 and 13 g/dL. They were not included in case of bone marrow disease or ongoing treatment that impairs erythropoiesis (such as chemotherapy…), uncontrolled hypertension, recent intravenous infusion of iron (within 1 week), blood transfusion within 1 week before inclusion, or preoperative blood transfusion already scheduled, the patient cannot be transfused or has refused consent for a blood transfusion, bedridden or very dependent patient. Demographic data (including gender, height and weight), medical history, usual medication, hip fracture details (i.e., intra- or extracapsular fracture) were recorded. Biological data were also obtained at study inclusion: hemoglobin, creatinine, transferrin saturation, ferritin, and C-reactive Protein (CRP) level. Patient questionnaire regarding post-operative rehabilitation, including his ability to walk a 10 ft distance without human assistance, number of hospitalization days in the month following surgery, and the date of home return (if it happens) was collected at 30 days following inclusion.

### Questionnaires

Patient’s QOL was evaluated at three-time points: inclusion (patient were asked about their quality of life before hip fracture when they were just admitted to the hospital for hip fracture), then 30 and 90 days after inclusion. We choose to use the 90 days results, to minimize the effect of hip fracture management on patient’s QOL assessment and because the assessment was centralized.

At 90-days, questionnaires were obtained by structured telephone interviews, centralized by one center (the CHU Angers) and done by a single trained interviewer, using a standardized and protocolized interview. When the patient could not answer, either the next of kin or the patient’s nurse answered all eligible questions. The following questionnaires were used:

- EuroQOL-5 dimensions 3 levels (EQ-5D) (11): patients were asked to indicate their state of health by selecting the most appropriate statement in each dimension (i.e., mobility, autonomy, activity, pain, and anxiety), a higher score being indicative of a poorer condition. In addition, the patient was asked to rate his/her health status on a scale from 0 to 100 (100 being the best health status the patient could imagine).
- Perceived Quality of life (PQOL). We used the summary variable (i.e., item number 20) of the 20-items PQOL questionnaire (12). This item evaluates patient’s happiness by asking “how happy are you?: very happy, happy, unhappy, very unhappy, or any of these answers.”
- The Instrumental Activities of Daily Living (IADL) (13). Patients were asked about their ability to use phone, to move, to take their medications and to take care of their budget. Each dimension was coted 0 (inability) or 1 (capable of). A global IADL score (from 0 to 4) was also calculated by adding each separate score.

### Data analysis

We compared groups of patients before and during the COVID period. A descriptive analysis of the patient’s characteristics was performed. Continuous variables were reported using the mean ± Standard Deviation (SD) or median [Q1-Q3], according to the distribution of the data. The normality was evaluated graphically. For parametric data, T-test was used and Kruskall-Wallis tests for non-parametric data. Categorical variables are presented as *n* (%), for the number with sample percentage. We compared those variables using Chi-square test.

We mainly focused on the quality of life evaluated with the EQ-5D score and performed multivariate analysis only for the five components of this score. We used ordinal regression to model the association between the ordinal response variable (i.e., each component of the EQ-5D score) and prognostic factors of altered quality of life. We obtained proportional odds ratios (OR) representing the associations of the period (before versus during COVID) on the likelihood of having poorer evaluations on the EQ-5D components. The OR values account for each increase of EQ-5D component levels and are represented with their 95% confidence interval (95%CI). The empirically chosen prognostic factors were age, gender status, the study site, the type of surgery, and the place of living when filling out the questionnaire. To evaluate the impact of containment and liberty restriction, we performed a sensitivity analysis by comparing the patients interviewed during periods with liberty restriction (containment periods) to those interviewed when only social distancing measures were applied (non-containment periods). For all analyses, a value of *p* < 0.05 was considered statistically significant. All analyses were performed with R software (version 3.6.3).

## Results

During the study period, 161 patients were included before the COVID and 213 during the COVID period, including 122 (57%) patients during COVID with containment periods and 91(43%) during COVID without containment periods (see Figure 1 for details). The patient’s characteristics were similar during the two periods (before/after COVID). Only 5(2%) COVID cases were diagnosed (i.e., with a positive PCR test) during their follow-up. Table 1 summarizes the patient’s characteristics during the two periods. Table 2 shows the answers to the EQ-5D questionnaire before and during the COVID period. During the COVID period, EQ-5D showed better patient pain/discomfort and anxiety/depression levels 90 days after a hip fracture. The PQOL happiness was not different, with around 81% of the patient being “happy” or “very happy” during the two periods (Table 3). The IADL was also similar during the two periods, but fewer patients could not manage their budget in the COVID period (Table 3).

**Table 1:** Patient’s characteristics during the two periods.

	Before COVID (*n* = 161)	COVID period (*n* = 213)	*p*
Age (years)	84 ± 9.5	84 ± 10	0.94
BMI (kg/m^2^)	23.5 ± 5.0	23.4 ± 4.4	0.89
Women	128 (79)	154 (72)	0.14
Extra-capsular fracture	85 (53)	101 (47)	0.36
Hypertension	98 (61)	138 (65)	0.50
Diabetes	25 (16)	42 (20)	0.36
COPD	12 (8)	19 (9)	0.75
Stroke	16 (10)	27 (13)	0.51
COVID +	*NA*	5 (2)	
Creatinine (μmol/L)	71 ± 33	79 ± 46	0.08
Hemoglobin (g/dL)	11.4 ± 1.2	11.5 ± 1.1	0.37
Death	19 (12)	17 (8)	0.29
Localization at D90			0.22
Previous place of residence	113 (81)	159 (85)	
Convalescent center	18 (13)	23 (12)	
Hospital	6 (4.3)	6 (3)	
Other/unknown	5 (3)	8 (4)	

Data are presented as mean ± SD or *n* (%). BMI, body mass index; COPD, chronic obstructive pulmonary disease; D90, 90 days.

**Table 2:** EQ-5D questionnaire answers before and during COVID periods.

	Before COVID (*n* = 161)	COVID period (*n* = 213)	*p*
Mobility			0.03
no problem	56 (40)	98 (52)	
some problem	73 (53)	70 (37)	
confined to bed	10 (7)	19 (10)	
Self-care			0.36
no problem	59 (42)	72 (38)	
some problem	54 (39)	87 (47)	
unable to wash or dress	26 (19)	28 (15)	
Usual activities			
no problem	53 (38)	62 (33)	0.38
some problem	62 (45)	98 (53)	
unable to perform	24 (17)	27 (14)	
Pain/discomfort			
no pain/discomfort	67 (49)	125 (67)	0.001
moderate pain/discomfort	56 (40)	55 (29)	
extreme pain/discomfort	15 (11)	7 (4)	
Anxiety/depression			
no	71 (51)	112 (61)	<0.001
moderate	45 (33)	67 (36)	
extreme	22 (16)	6 (3)	
EQ-5D index (/100)	61.4 ± 22.6	67.4 ± 18.1	0.07

Data are presented as mean ± SD or *n* (%). The number of answers obtained varied across the questions, the percentage is calculated according to the total of answers obtained for the each specific questions.

**Table 3:** PQOL and IADL questionnaire answers before and during COVID periods.

	Before COVID (*n* = 161)	COVID period (*n* = 213)	*p*
PQOL happiness			0.922
very sad	4 (7)	3 (5)	
sad	7 (11)	9 (14)	
happy	41 (67)	44 (9)	
very happy	9 (5)	8 (12)	
IADL			
unable to use the phone	10 (9)	16(9)	1
unable to use transportation	26 (27)	34 (26)	0.987
unable to handle his/her medication	53 (44)	77 (50)	0.409
unable to manage finance	26 (27)	16 (14)	0.019
Total IADL score (/4)	3.2 ± 1.3	3.2 ± 1.1	0.946

Data are presented as mean ± SD or *n* (%). The number of answers obtained varied across the questions, the percentage is calculated according to the total of answers obtained for the each specific questions.

In the multivariate analysis, we found the same results, with increased odd ratios of having poorer outcomes before COVID for pain/discomfort (OR 2.38, 95%CI [1.41–4.15], *p* = 0.001), for anxiety (OR 1.89 [1.12–3.21], *p* = 0.017) and for mobility (1.69 [1.02–2.86], *p* = 0.044), but not for self-care (OR 1.22 [0.72–2.08], *p* = 0.47) and usual activity (OR 0.99 [0.60–1.66] *p* = 0.98).

**Figure 2:**
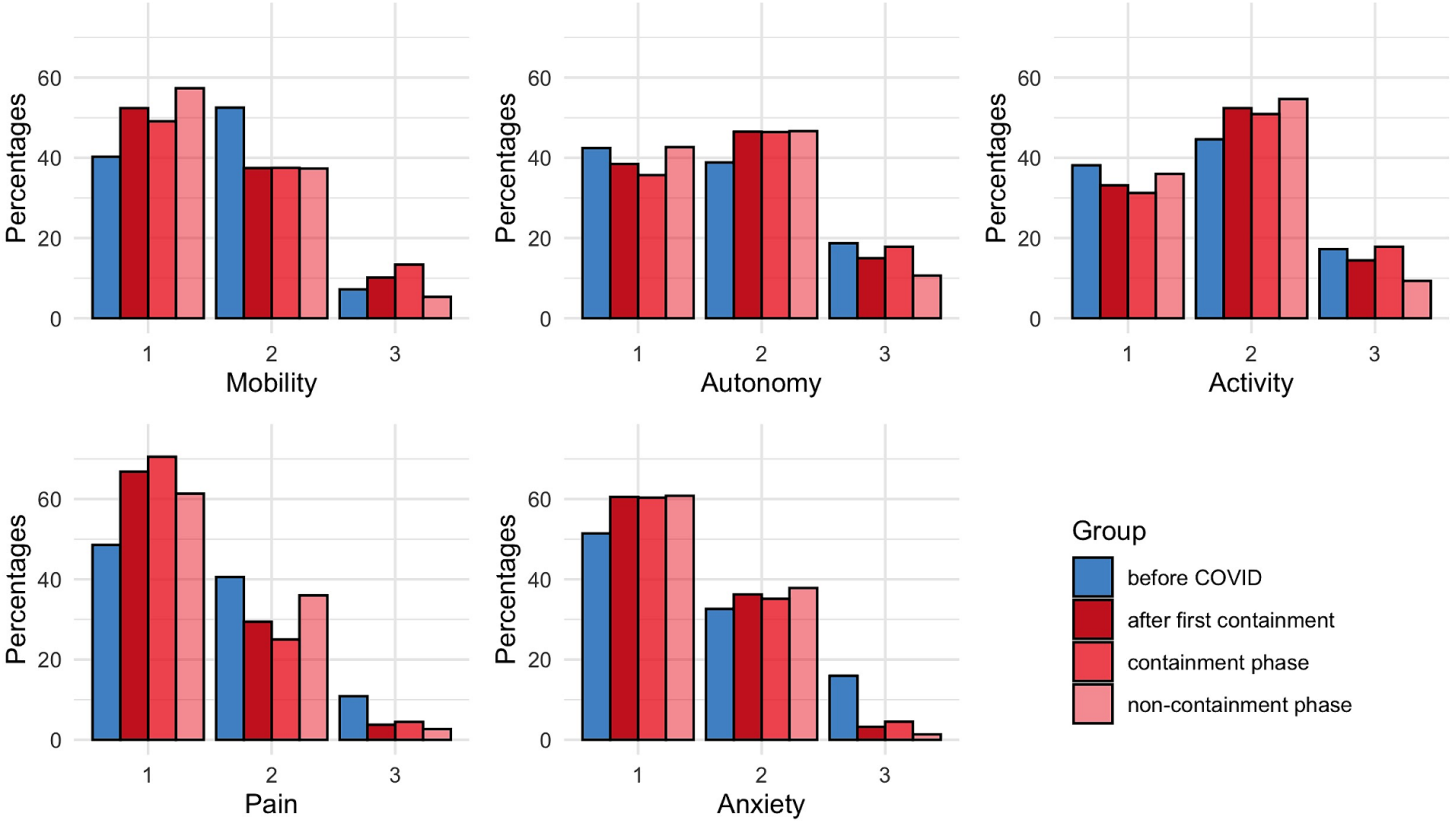
Distribution of EQ-5D scores measured at D90 for each domains according to the periods. Blue bars represent values obtained before COVID. Red bars show values obtained during COVID periods: dark red for all the COVID periods, light red during containment phases only and pink bars during non-containment phases only. A higher score is indicative of a poorer condition for each domain.

Because the public rules changed during the COVID period, we made a sensitivity analysis by comparing the patients interviewed during periods with liberty restriction (containment periods, *n* = 122) to those interviewed when only social distancing measures were applied (non-containment periods, *n* = 91). The two populations were similar and not different from the before COVID period (Table 4). We found no difference in EQ-5D answers between the containment and non-containment periods (Figure 2; Table 5). There were also no differences in PQOL and IADL scores (Table 6). However, in both groups, we found better EQ-5D scores for pain/discomfort and anxiety/depression 90 days after a hip fracture, compared to before COVID period (Figure 2).

**Table 4:** Patient’s characteristics during COVID period with or without containment.

	Without containment phase (*n* = 91)	Containment phase (*n* = 122)	*p*
Age (years)	84 ± 10	84 ± 10	0.58
BMI (kg/m^2^)	23.6 ± 4.5	23.3 ± 4.4	0.68
Women	60 (66)	94 (77)	0.10
Extra-capsular fracture	46 (51)	55 (45)	0.56
Hypertension	56 (62)	82 (67)	0.48
Diabetes	19 (21)	23 (19)	0.85
COPD	7 (8)	18 (15)	0.76
Stroke	9 (10)	27 (13)	0.40
Creatinine (μmol/L)	80 ± 34	78 ± 53	0.79
Hemoglobin (g/dL)	11.7 ± 1.1	11.4 ± 1.1	0.11
Death	8 (9)	9 (7)	0.84
Localization at D90			0.93
Previous place of residence	65 (86)	94 (84)	
Convalescent center	10 (13)	13 (12)	
Hospital	1 (1)	5 (5)	
Unknown	7 (8)	1(1)	

Data are presented as mean ± SD or *n* (%). BMI, body mass index; COPD, chronic obstructive pulmonary disease; D90, 90 days.

**Table 5:** EQ-5D questionnaire answers during COVID period with or without containment.

	Without containment phase (*n* = 75)	Containment phase (*n* = 112)	*p*
Mobility			0.18
no problem	43 (57)	55 (49)	
some problem	28 (37)	42 (38)	
confined to bed	4 (5)	15 (13)	
Self-care			0.35
no problem	32 (43)	40 (36)	
some problem	35 (47)	52 (56)	
unable to wash or dress	8 (11)	20 (18)	
Usual activities			
no problem	27 (36)	35 (31)	0.26
some problem	41 (55)	57 (51)	
unable to perform	7 (9)	20 (18)	
Pain/discomfort			
no pain/discomfort	46 (61)	79 (71)	0.25
moderate pain/discomfort	27 (36)	28 (25)	
extreme pain/discomfort	2 (3)	5 (5)	
Anxiety/depression			
no	45 (61)	67 (60)	0.48
moderate	28 (38)	39 (35)	
extreme	1 (1)	5 (5)	
EQ-5D index (/100)	63.8 ± 17.7	70.1 ± 18.1	0.13

Data are presented as mean ± SD or *n* (%).

**Table 6:** PQOL and IADL questionnaire answers during COVID period with or without containment.

	Without containment phase (n = 67*)	Containment phase (n = 105*)	*p*
PQOL happiness	*n = 25*	*n = 39*	0.49
very sad	2 (8)	1 (3)	
sad	2 (8)	7 (18)	
happy	17 (68)	27 (69)	
very happy	4 (16)	4 (10)	
IADL			
unable to use the phone	3 (4)	13 (12)	0.11
unable to use transportation	9 (17)	25 (33)	0.06
unable to handle his/her medication	31 (48)	46 (52)	0.82
unable to manage finance	8 (16)	8 (12)	0.64
Total IADL score (/4)	3.3 ± 1.1	3.1 ± 1.1	0.24

Data are presented as mean ± SD or *n* (%). The number of answers obtained varied across the questions, the percentage is calculated according to the total of answers obtained for the each specific questions. * Maximum number of answers for a question.

## Discussion

In this ancillary study of the HiFIT randomized controlled study, we observed that patient’s quality of life measured using different scales was not altered during the COVID period compared to before COVID, 90 days after a hip fracture. Surprisingly, the Pain/Discomfort and Anxiety dimensions of the EQ-5D questionnaires were even better during the COVID period.

One may have expected that during the COVID periods, marked by increased mortality of the older adult (1) and many liberty restrictions (including confinement, cover-few, social-distancing measures…), the QOL of patients with a hip fracture would have been altered. Indeed, several studies have demonstrated an impaired QOL in the older adult faced with COVID-19. In a Canadian longitudinal study, in which 104 active older adult persons were asked about their QOL before and during the COVID-19 period (twice, during lockdown (*n* = 94) and confinement periods (*n* = 86)), the lockdown period was associated with a significant decrease in perceived health (−6.3 ± 15/100) and in QOL (in mean − 1.1 over 25) (14). Interestingly, the perceived health status improved for some patients. In addition, there was no difference between the two periods of lockdown (with around 9 months between them), indicating a possible adaptation of this population (14). We also did not find any difference between the lockdown and the non-confinement periods (several weeks after the initial lockdown). In another cohort of Portuguese people under quarantine, responders (*n* = 915) had only a slight decrease in their QOL (measured using the EQ-5D, with mean global health of 86.1 ± 2.7), compared to the Portuguese population prior to the COVID-19 (88.7 ± 0.5) (15, 16). However, the proportion of older adult patients was small in that study (13% were 60 years old or more). In a cohort of 39 older US patients, the “Stay home, Stay healthy order” period was associated with significantly more anxiety symptoms and less satisfaction with participation in their social roles than prior to the COVID-19 period (17). Surprisingly, patients in this cohort reported less fatigue during the “Stay Home” period, and some of them found positive aspects of being home, such as deeper connections (often digital) with their loved ones or time to engage in new hobbies (17). In a large cohort of 3,041 patients from Spain (issued from 4 different cohorts of patients >60 or > 65 years old), changes in mental and physical health were variable across the different cohorts, with some participants showing an increase (mean 4.79 points) in the PCS of the SF-12 and a slight decrease in the MCS (−1.19 points in mean). Older patients may have more capacity to face the pandemic. Indeed, a US-based study found that older adults reported better mental health than younger at the beginning of the COVID-19 pandemic (18). We studied a specific population of older adult patients suffering from hip fracture, which is known to impact patient’s QOL. Indeed, the lack of exercise impacts QOL in the older adult (19), maybe more than COVID-19 did (5). The patients may have put the hip fracture impact on QOL more into perspective during the COVID period.

In our cohort, only 2 patients were diagnosed as COVID positive. This could also explain the absence of poorer outcomes in the COVID period. Indeed, it has been reported that COVID-19 infection is associated with a decrease in QOL in the older adult. In a longitudinal prospective Spanish cohort study, COVID-19 infection has been associated with a mean reduction of 30 points in the Barthel index (from 83.2 ± 15.2 to 52.3 ± 27.22 pre and post-COVID-19 infection mean values) with a significant decrease in the 10 domains investigated (20).

We did not use specific questionnaires to assess mental health. In a recent review, it has been shown that COVID-19 is associated with an impact on mental health, with depressive symptoms in almost half of the older adult patients (21). However, we found no signal of poorer condition in the items evaluating mental health in the questionnaires we used (neither in the EQ-5D anxiety/depression item nor in PQOL question). This could be due to the usual impact of hip fracture, which could be more critical in the “usual” conditions than during the pandemic. The prevalence of depression is around 23% in this population (22). Indeed, the limitation of physical activity is known to negatively impact QOL and mental health (23). It is thus possible that the usual impact of hip fracture was lesser in that context. The level of resilience is high in patients with hip fracture, with a high level for 30% of patients and a normal level for 45%, as observed in an analysis of three cohorts (total of 541 patients) (24).

Our study has some more limitations. Indeed we could not evaluate the same patients before and after the COVID period. We thus cannot directly evaluate the impact of the COVID period on QOL. We do not have the data to evaluate the quality of recovery after the hip fracture, which could impact the QOL. However, it should be equivalent during the two periods. Many scores and questionnaires are available to evaluate the QOL, we chose some of them, usually proposed in this older adult population, but many more could have been proposed. At last, the patients did not answer all the questions, so the power of the study may be limited for some items, notably the PQOL.

The restrain in liberty was thus well supported in this older adult population, which is supposed to benefit the most from containment measures. This may encourage public authorities to use them again in event of a new pandemic. “Stay home” policies may thus be proposed again, especially for the older adult patients, in the event of a new pandemic.

## Conclusion

In conclusion, we observed that the COVID period, with liberty restrictions, was not associated with a poorer quality of life in older adult patients 3 months after a hip fracture. Pain, anxiety and mobility were even better in the COVID period. This may encourage public authorities to use “stay home” policies for older adult patients, in case of a new pandemic.

## Data availability statement

The raw data supporting the conclusions of this article will be made available by the authors, without undue reservation.

## Ethics statement

The studies involving humans were approved by Comité de Protection des Personnes Ouest II, number 2016/42, approval date November 15, 2016. The studies were conducted in accordance with the local legislation and institutional requirements. The participants provided their written informed consent to participate in this study.

## Author contributions

SL: Conceptualization, Formal analysis, Funding acquisition, Investigation, Methodology, Supervision, Writing – original draft, Writing – review & editing. XC: Investigation, Writing – review & editing. BB: Investigation, Writing – review & editing. ML-C: Investigation, Writing – review & editing. VC: Investigation, Writing – review & editing. NG: Investigation, Writing – review & editing. TL: Investigation, Writing – review & editing. ER: Investigation, Writing – review & editing. ML: Data curation, Formal analysis, Investigation, Methodology, Writing – review & editing.

## Group members of the HiFIT study group (and study enrolment sites)

CHU Angers, Guillaume BOUHOURS, Sigismond LASOCKI, Adeline LEBAIL, Maxime LEGER, Elsa PAROT-SCHINKEL, Emmanuel RINEAU, Louis RONY, Bruno VIELLE; CHU – Hôpital Lapeyronie Montpellier, Xavier CAPDEVILLA, Thibault LOUPEC, Benjamin MOUNET, Fabien SWISSER; HIA Clermont-Tonnerre, Marc Danguy des Deserts; CHU Nantes, Raphael Cinotti, Nicolas Grillot, Karim Asehnoune, Antoine Roquilly; CHU de Rennes, Hélène Beloeil, Maria Lahlou-Casulli; Medipôle, Lyon Villeurbanne, Vincent Collange, Sébastien PARENT; Department of Anesthesiology and Intensive Care medicine, Ramsay Sante, Sauvegarde Clinic, Lyon, France, Bertrand DELANNOY, Olivier Desebbe; CHD Vendée, Alexis Duchalais; CHU Poitiers, Bertrand DRUGEON, Jérémy GUENEZAN; CHU Grenoble Alpes, Pierre BOUZAT, Sabine DREVET, Gaetan GAVAZZI, Jules GREZE; CHRU Lille, Benjamin BIJOK, Delphine GARRIGUE; Hospices Civils de Lyon, Lyon, France, Jean-Stéphane DAVID.
